# Conceptual metaphors and image construction of China in the space probe reports of *China Daily*: a social cognitive approach

**DOI:** 10.3389/fpsyg.2023.1202988

**Published:** 2023-06-08

**Authors:** Xueying Li, Danyun Lu

**Affiliations:** College of International Studies, National University of Defense Technology, Changsha, China

**Keywords:** conceptual metaphor, national image, space probe, image construction, *China Daily*

## Abstract

The success of Shenzhou XIII and Chang'e-5 mission became a milestone in China's aerospace history and represented China's latest attempt to contribute to international space industry, which greatly promoted the China's national image. However, rare studies have examined the image construction in aerospace field. Thus, this study takes conceptual metaphors as the guiding theory and studies conceptual metaphors in China Daily news release on Chang'e-5 and Shenzhou XIII from 2008 to 2021. It focuses on the types of metaphors used, the semantic features of the metaphors, and the characteristics of Chinese images in aerospace field. It is found that China Daily widely uses conceptual metaphors in its news release on space probe, which mainly includes 11 conceptual metaphor categories such as “endeavor,” “great significance,” “time” and “journey,” and 20 types of conceptual metaphor subcategories, all of which are working together to construct the image of China in aerospace industry, which is characterized with the following features: a dream-building action with lofty goals, an enterprising action which represents the prosperity and progress of China, an exploratory action that is constantly forging ahead and pursuing, a leading action that opens a new chapter and leads a new journey, a braving action which dares to be the first to live in the space, and an achieving action to create a community with a shared future for mankind.

## Introduction

The Chinese space probe program began in the 1950s and launched the first satellite on April 24, 1970. After that, in 1971, manned space engineering was propounded. In 1992, according to the “three-step” development strategy, China's manned space project was implemented, and till 2010, the manned space station project was officially launched. On October 16, 2021, the Shenzhou XIII mission launched on a Long March 2F carrier rocket and started the longest manned flight in space.

On November 22, 2000, the State Council issued the white paper “China's Aerospace,” which put forward the goal of space exploration focusing on lunar exploration. Two years later, a national cooperative engineering system framework was established to improve the carrying capacity and meet the needs of deep space exploration. It included a three-step lunar exploration program (“orbit, land, and return”) named Chang'e lunar probe, which was projected to be completed in 2020. On November 24, 2020, China launched the Chang'e-5 spacecraft to collect and return samples from the moon, which was the first space expedition from any country to bring back lunar samples in decades.

The success of the two missions became a milestone in China's aerospace history and represented China's latest attempt to contribute to the international space industry, which greatly promoted China's national image. However, rare studies have examined image construction in the aerospace field. At present, scholars greatly concentrate on the construction of national image, and its linguistic construction has also attracted special attention (Sun, 2009), especially the cognitive linguistic construction (Hu, 2011; Li, 2011). Among them, conceptual metaphor is a commonly used technique (Liang, 2013, p. 113). Therefore, by undertaking the analysis, the current study has two specific goals: (1) to investigate the mechanism of the conceptual metaphors in the case of space probes by the series reports of *China Daily*, and (2) to explore the national image of China that is constructed by these metaphorical words in the aerospace news reports.

The significance of this study lies in investigating the conceptual metaphors used in the news series of space probe. First, it identifies the metaphor words in the news texts and summarizes the semantic features of these metaphors. Then, based on these semantic features, it systematically examines the national image of China constructed by the aerospace news reports. Finally, the result of this project will also provide a reference for journalists to know and improve the national image in future news reports.

## Literature review

### Literature review of national image

Initial studies of images can be traced back to the work of Kenneth Boulding (Davies et al., 2021, p. 70), who regards image as “the subjective knowledge of the world which governs people's behavior” from the aspect of the international political field (Boulding, 1956, p. 5–6). Nimmo and Savage (1976) extended the meaning of “image” and believed that image is a human construct imposed on an array of perceived attributes projected by an object, event, or person. Every object has its image, as well as an image perceived by others. Individuals, organizations, and even countries have images. These images are of vital importance since they influence the process through which one deals with others. For a country, this image impacts many arenas, such as the military, politics, economics, and tourism, to name but a few.

For the definition of the national image, Martin and Eroglu (1993) thought that national image refers to the combination of all descriptive, inferential, and informational faith that a person has toward a particular country. And that impression is mainly reflected in the aspects of politics, economy, and science and technology. Kunczik (1997) argued that national image is the representation that a person holds of a given country, what a person believes to be true about a nation and its people. The domestic scholar (Sun, 2002) pointed out that national image refers to the external and internal public's understanding and evaluation of a particular country in terms of its politics, economy, society, culture, and geographical conditions. The national image is fundamentally determined by the comprehensive national strength of the country, but it cannot be simply equated with the actual condition of that country. National image is the comprehensive reflection of a country's soft power and hard power, thus, there is no doubt that national image can be constructed from many aspects.

At present, the construction of a national image has attracted increasing attention, and its language construction has also attracted the special attention of scholars (Sun, 2009, p. 60–83). In foreign linguistic field, Doorslaer (2010) discussed the construction of national image in the translation of news terms in new media using the theory of iconology; Young (2012), based on the Hofstede's five cultural dimensions, conducted a qualitative research on Russian culture from the perspectives of the Russian people, government, culture, tourism and products, and discussed how the state should take “identity as the national image”; Lee and Kim (2013) made some analysis on how the translation of the South Korean tourism influenced the national image; Adeyemi (2017) studied semiotic resources in resisting Nigeria's negative stereotypes in the international community, especially the country's national image in the Western media; Pieta (2018) demonstrated that Poland was portrayed as both a friend and an enemy when its literary texts was translated into Portuguese and investigates how the role of different versions of literary texts influenced the construction of the national image.

In the domestic linguistic field, the studies on national image mainly focus on constructing the image of China and various research approaches have been used, such as the translation strategy (Hu, 2010, 2014; Wang, 2012; Lu, 2013; Yuan, 2014), multimodal discourse analysis (Pan and Zhang, 2013), cognitive analysis (Liang, 2013; Liu, 2017), corpus linguistics (Hu and Li, 2017; Yu and Chen, 2019), rhetoric theory (Hu and Xue, 2010), critical discourse analysis (Liu and Yu, 2014; Li, 2017; Pan and Dong, 2017), narrative analysis (Ren, 2017; Zhu et al., 2019), and cultural discourse analysis (Wang, 2017). For the examination of perceived national images, we can see that the expression of a national image widely depends on different linguistic devices. Among them, metaphor deserves our attention (Liang, 2013, p. 115).

### Literature review of national image and metaphors

Human thought processes are largely metaphorical. This is what we mean when we say that the human conceptual system is metaphorically structured and defined. Metaphors as linguistic expressions are possible precisely because there are metaphors in a person's conceptual system. In addition, conceptual metaphors are grounded like our everyday interaction with the world. That is, conceptual metaphor has an experiential basis (Lakoff and Johnson, 1980, p. 7, 9; Vyvyan and Melanie, 2006, p. 295). Thus, it can be seen that metaphorical linguistics are reflections of an underlying conceptual association based on people's daily experiences. According to cognitive semanticists, metaphor is a conceptual mapping between the source and target domain. The source and target domains are always unidirectional. Kövecses (2002; p. 20) put it, “target domains are abstract, diffuse and lack clear delineation; as a result, they ‘cry out' for metaphorical conceptualization”, and found that the most common source domains for metaphorical mappings include domains relating to the HUMAN BODY (the heart of the problem), ANIMALS (a sly fox), PLANTS (the fruit of her labor), FOOD (he cooked up a story), and FORCES (don't push me!). The most common target domains included conceptual categories like EMOTION (she was deeply moved), MORALITY (she resisted the temptation), THOUGHT (I see your point), HUMAN RELATIONSHIPS (they built a strong marriage), and TIME (time flies). According to Raymond and Gibbs (2011), the range of abstract conceptual domains that appear to be structured in some manner by conceptual metaphor is immense and includes emotions (Kovecses, 2000), the self (Lakoff and Johnson, 1999), morality (Johnson, 1993), politics (Lakoff, 1996; Musolff, 2004), science concepts (Brown, 2003; Larson et al., 2006), illness (Sontag, 1978; Gibbs and Franks, 2002), psychoanalytic concepts (Borbely, 2004), legal concepts (Winter, 2002), mathematics (Lakoff and Núñez, 2002), and certain cultural ideologies (Goatly, 2007). Thus, this study mainly adopts the domains, such as cultural ideologies (traditional culture), morality (strivers, share, and leadership), time (time), human relationships (family), journey (journey), and some other abstract concept domains (music, chapter, significance, and difficulty) to contract the metaphor typologies.

In recent examples of scholarship on national image and metaphors, Liang (2013) investigated the special role of the metaphor in image construction, sorted, and analyzed the collected corpus of a large number of news reports referring to the country's name. It is found that in the current news report discourse construction of the national image, the shaping of the national image of conceptual metaphor at the cognitive level was mainly reflected in four aspects, namely, animalization and personification, solidification and temporary, nationality and ideology. Pan and Zhang (2013) identified and interpreted metaphor and metonymy in *China's national image promotion: perspectives*, analyzed the interaction mode between them, discussed their different roles in forming cohesion and coherence of multimodal discourse, and revealed the cognitive mechanism of multimodal discourse. Guan (2018) made a systematic study on how to construct the Russian national image in the Russian national image propaganda film based on the theory of multimodal metaphor. Liang (2018), using the theory of framing theory and conceptual metaphor, analyzed 124 reports in the China column of the *Economist* in 2016, and discussed the attitude of British media toward China's economy. Fitnat (2021) researched the use of metaphors among students and teachers to determine the images they had in their minds regarding a specific set of developed countries. It was concluded that there is a strong relationship between metaphor creation and age, and that metaphor production increases rapidly as the students get older and more knowledgeable. It was also determined that metaphor quality and production rate decreases as teachers' professional seniority increased. Musolff (2021) explores the nation as a body metaphor in the specific cultural context and concluded that “Scenarios thus serve as the background against which the new concept or argumentative application can be analyzed in relation to the historically situated communicative context”.

However, research on the construction of the national image in the aerospace field using the conceptual metaphor theory is rare. Only one recent example of study, Li (2016), based on the theory of conceptual metaphor, examines the characteristics of metaphor mechanism in the news reports in the case of space probes and analyzes the changes of metaphor used in different social and historical backgrounds. Studies on the construction of the national image in the aerospace field are still rare. Hopefully, this article may lead to further research into this topic.

## Corpus and methodology

*China Daily* was established in 1981 as the national English-language newspaper. It serves more than 330 million readers all over the world and is a default choice for people who read about China in English.^1^ This article collects 460 news reports of Chang'e 5 and Shenzhou XIII as the corpus data. And then based on the conceptual metaphor theory, it identifies the metaphors used in these news reports, examines the semantic features of these metaphors, and investigates the national image of China constructed by *China Daily*.

### Data of the research

On the *China Daily* website, 330 news reports are collected with “Chang'e-5” as the subject keyword. The time selected is from September 24, 2008 to January 29, 2021. A total of 130 news reports are collected with “Shenzhou XIII” as the subject keyword. The time selected was from October 10, 2011 to April 16, 2022. The years 2008 and 2011 were the earliest times when *China Daily* reported on Chang'e-5 and Shenzhou XIII. The years 2021 and 2022 were the time that Chang'e-5 and Shenzhou XIII successfully completed the mission, respectively.

### Research questions

This article aimed to answer the following three questions:

(1) What conceptual metaphors are used in the news reports of Chang'e 5 and Shenzhou XIII in *China Daily*?
(2) What are the semantic features of these conceptual metaphors?
(3) What kinds of national image are constructed by the news series of *China Daily*?

### Criterion for metaphors

The metaphor was characterized by the schematic form: A is B, as in *Achilles is a lion*. As a consequence, metaphor has been identified since the time of Aristotle with implicit comparison. In other words, while metaphor is based on the comparison of two categories, the comparison is not explicitly marked (Vyvyan and Melanie, 2006, p. 293). Thus, in this article, if the concept in one domain can be expressed by the concept in another domain, we can suppose that the conceptual metaphor is produced. The explicit metaphor can be expressed as “XXX (one concept)” is “A”, otherwise, if the “XXX (one concept)” is replaced by “A”, the “A” is the implicit metaphor. In this article, the explicit metaphors refer to some examples such as “Chang'e-5 is one of the most complicated and challenging **missions** in China's aerospace history”; and the implicit metaphors include “TT&C missions of the **Shenzhou** and **Tianzhou** spacecraft series, **Tianhe** core module, **Chang'e** lunar probe series, and **Tianwen**-1 Mars probe have been completed successfully.”

## Data analysis

### Conceptual metaphor in the case of the chang'e-5 by series reports

The Antconc software was employed to extract the sentences with keywords. Such words as “Chang'e; lunar exploration; lunar sample; moon probe project” were treated as keywords and were used to search for the conceptual metaphor sentences in the corpus of 330 news reports. As a result, 506 statements using conceptual metaphors have been found. After identifying and sorting the conceptual metaphors in these sentences, the main conceptual domains and the number of metaphors are given in Table 1.

**Table 1:** Main conceptual domains and the number of metaphors.

**Domains**	**Sub-domains (5,135)**	**Words (occurrence)**	**Frequency (%)**
Traditional culture	Traditional culture (1,638)	Chang'e (1,323); Yutu/Jade Rabbit (129); Shenzhou (69); Tiangong (58); Tianwen (41); Queqiao (18)	31.90
Strivers	Mission (1,228)	Mission (712); exploration (454); attempt (21); prototype (21); endeavor (20)	23.91
	Goal (103)	Start (46); goal (42); dream (14); wake-up call (1)	2.00
	Spirit (15)	Spirit (15)	0.29
Significance	Achievements (624)	Success/successful/successfully (276); achieve/achievement (85); historic (82); breakthrough/breakthroughs (39); complete (35); strategic (30); feat (21); contribute (20); accomplish/accomplishment (17); fulfill (15); highlight (4)	12.15
	Power (181)	Lift (108); power (72); emphatic (1)	3.52
	Action(5)	Leap (5)	0.10
	Fruit(3)	Fruit (3)	0.06
Time	Time period(301)	Develop (295); upcoming (6)	5.86
	Point in time(32)	Era (17); moment (14); timeline (1)	0.62
Journey	Trip(656)	Long March (427); step (76); phase (71); stage (53); course (15); on its way/pave the way (14)	12.78
	Landmark (45)	Milestone (24); landmark (19); roadmap (2)	0.88
Difficulty	Difficulty (121)	Challenge/challenging (66); complicated (41); overcame (14)	2.36
Family	Family (69)	Home (39); backup (20); safely (8); homecoming (2)	1.34
Leadership	Leadership (62)	Heads (53); lead (9)	1.21
Share	Share (32)	Share (29); willingness (3)	0.62
Chapter	Chapter (16)	Chapter (16)	0.31
Music	Music (4)	Trilogy (2); rehearsal (2)	0.08

**Figure 1:**
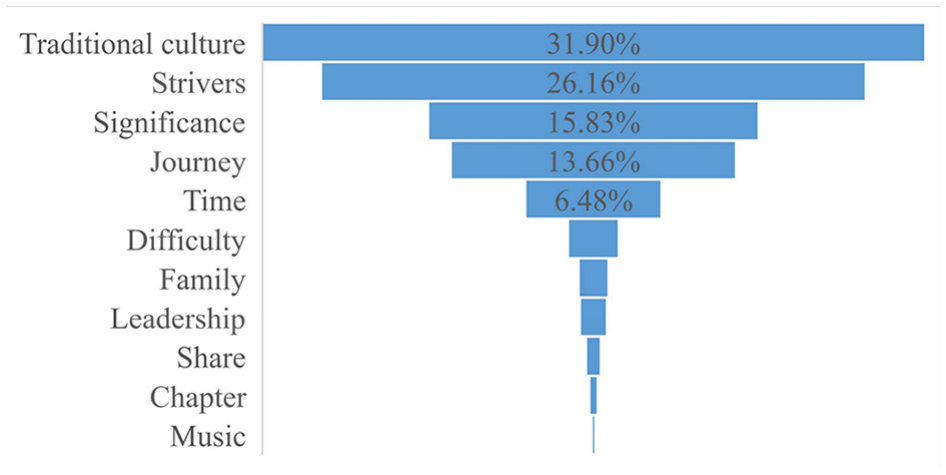
Macro-level semantic domains of conceptual metaphors.

**Figure 2:**
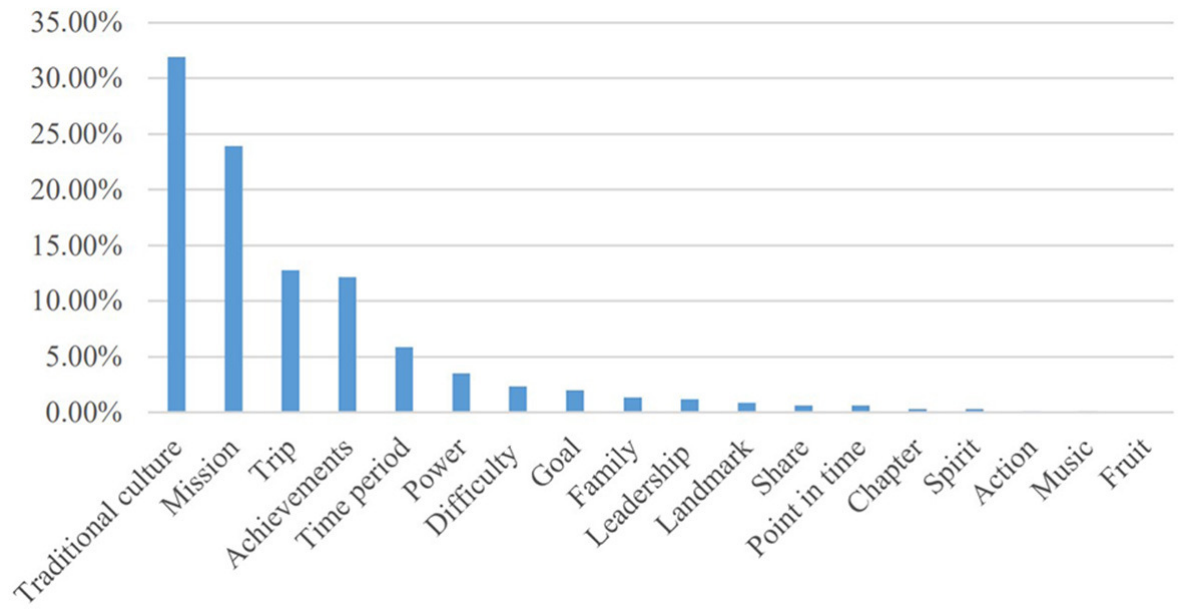
Micro-level semantic domains of conceptual metaphors.

The table and Figures 1, 2 above show that there are mainly 11 conceptual domains and 18 conceptual sub-domains in the series of news by the case of Chang'e-5 aerospace. The characteristics of these conceptual metaphors will be discussed as follows:

The conceptual metaphor domain “Traditional culture (Chang'e, Yutu/Jade Rabbit, Shenzhou, Tiangong, Tianwen, Queqiao)” is the most widely used metaphor in the series reports. These metaphors are from Chinese poems and myths, which express Chinese people's yearning for the moon and the spirit of exploration for the truth. Thus, this conceptual metaphor domain highlights that Chinese people look forward to exploring the moon since the ancient time.

“Strivers” is also found to be a key semantic domain in these news reports, in which “Mission” as the sub-domain is the most widely used metaphor, and the sub-domains “Goal” and “Spirit” also exist. The most widely used metaphor word is “mission”, which appears 712 times, and the “exploration” occurs 454 times. These metaphors emphasize that Chinese people advance bravely and modestly. The “Strivers” metaphor domain shows that China's aerospace industry is in the primary stage of rising and continuous exploration. The sub-domains “Goal (start, goal)” and “Spirit (spirit)” are positive, optimistic, and enterprising, which show that China is full of confidence in future for the space industry.

The conceptual metaphor “Significance” was ranked third, in which the conceptual metaphor “Achievement” is the key semantic domain, and the sub-domains “Power (lift, power, and emphatic),” “Action (leap),” and “Fruit (fruit)” are also found. The most widely used metaphors “success/successful/successfully” appear 278 times. These conceptual metaphors express the high evaluation of the completion of the space mission of chang'e-5, which has historic and strategic significance in China's space industry.

The conceptual metaphor domain “Time” includes sub-domains of “Time period (develop and upcoming)” and “Point of time (era, moment, and timeline)”. This conceptual metaphor domain evaluates the current achievements and future development of China's aerospace industry. It shows that chang'e-5 is of great significance to China's aerospace industry and has made great contributions in promoting the development of China's aerospace industry. The conceptual metaphor domain “Journey” includes sub-domains of “journey (Long March, step, phase, stage, course, and on its way/pave the way)” and “Landmark (milestone, landmark, and roadmap)”, which shows that China's aerospace industry is still “on the way” and has a lot of room for development. The sub-domain “road sign” is symbolic and directional, which shows that the successful completion of the mission of Chang'e-5 is of great symbolic significance in China's aerospace history and plays a directional role in the development of the aerospace industry in future.

It also uses the conceptual metaphor domain “Difficulty (challenge/challenging, complicated, and overcame)”, which expresses that the Chang'e-5 is complex, challenging, and a big breakthrough in China's technology. The words in the “Family (home/backup/safely/homecoming)” domain are warm and concerned. It marks that China contains humanistic feelings even in the technological field. The “Leadership (heads and lead)” domain shows that the aerospace industry in China has been at the forefront of the world and leads the development of aerospace science in the world. The “Share (share/willingness)” domain points out that China is willing to share the technological fruit with other countries and builds a community of shared future for mankind in the aerospace field. The last two domains “Chapter” and “Music (trilogy and rehearsal)” investigate that Chang'e-5 greatly promotes the development of the Chinese aerospace industry and China has a good wish for the space industry.

Next, the conceptual metaphor domains can be divided into three categories, and domains in each category and their word frequency are listed as follows:

1. Chinese aerospace industry is going upward (2,079): Chang'e-5 is mission (1,228) > Chang'e-5 is journey (656) > Chang'e-5 is goal (103) > Chang'e-5 is landmark (45) > Chang'e-5 is point of time (32) > Chang'e-5 is spirit (15).
2. China has a good wish for the space industry (1,743): Chang'e-5 is traditional culture (1,638) > Chang'e-5 is family (69) > Chang'e-5 is share (32) > Chang'e-5 is music (4).
3. Chang'e-5 is of great significance (1313): Chang'e-5 is achievement (624) > Chang'e-5 is time period (301) > Chang'e-5 is power (181) > Chang'e-5 is difficulty (121) > Chang'e-5 is leadership (62) > Chang'e- 5 is chapter (16) > Chang'e-5 is action (5) > Chang'e-5 is fruit (3).

### Conceptual metaphor in the case of the Shenzhou XIII by series reports

The Antconc software was also employed to extract the sentences with keywords. Such words as “Shenzhou, astronauts/taikonauts, and space industry” were treated as keywords and were used to search for the conceptual metaphor sentences in the corpus of 130 news reports. As a result, 437 statements using conceptual metaphors have been found. After identifying and sorting the conceptual metaphors in these sentences, the main conceptual domains and the number of metaphors are given in Table 2.

**Table 2:** Main conceptual domains and the number of metaphors.

**Domains**	**Sub-domains (835)**	**Words**	**Frequency (%)**
Traditional culture	Traditional culture (520)	Shenzhou (295); Tiangong (108); Tianhe (52); Tianzhou (48); Tianwen (7); Wentian (3); Mengtian (3); Chang'e (3); culture (1)	62.28
Strivers	Mission (153)	Mission (144); exploration (9)	18.32
	Goal (2)	Spirit (1); proactive (1)	0.24
	Spirit (1)	Dream (1)	0.12
Significance	Meaning (57)	Success/successful/successfully (22); complete (21); remarkable (2); historic (2); improve/improvement (4); revolutionary (1); strategic (1); advanced (1); promote (2); cutting-edge (1)	6.83
	Achievement (26)	Accomplish/accomplishment (17); achieve/achievement (5); feat (1); contribute (2); tribute (1);	3.11
	Action (3)	Stride (2); seize (1)	0.36
Journey	Trip (53)	Long March (27); journey (13); travel (4); trip (4); voyage (1); level (1); foundation (2); closed the gap (1)	6.35
	Occupants (2)	Occupants (2)	0.24
	Landmark (1)	Milestone (1)	0.12
Family	Family (10)	Home (5); backup (5)	1.20
Leadership	Lead (3)	Leading (1); guidelines (1); forerunners (1)	0.36
	Hero(3)	Hero (3)	0.36
Time	Point in time(1)	Age (1)	0.12

**Figure 3:**
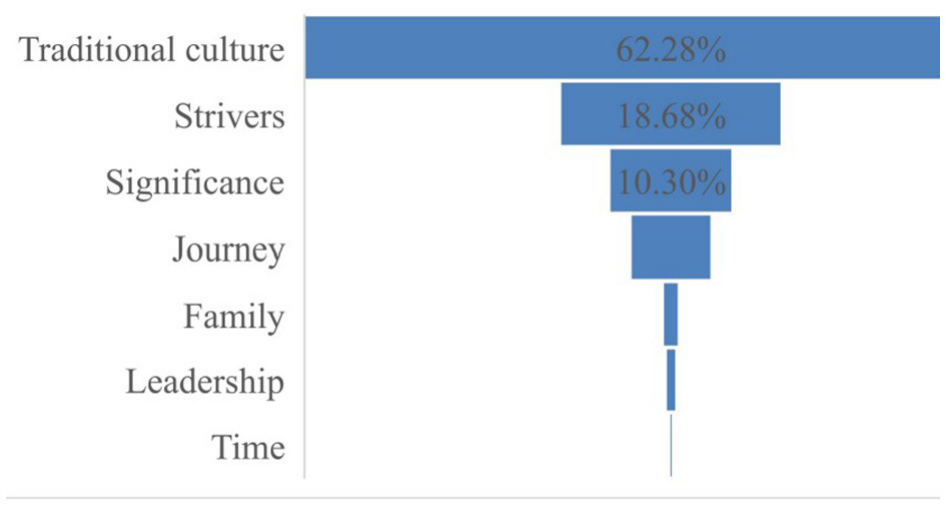
Macro-level semantic domains of conceptual metaphors.

**Figure 4:**
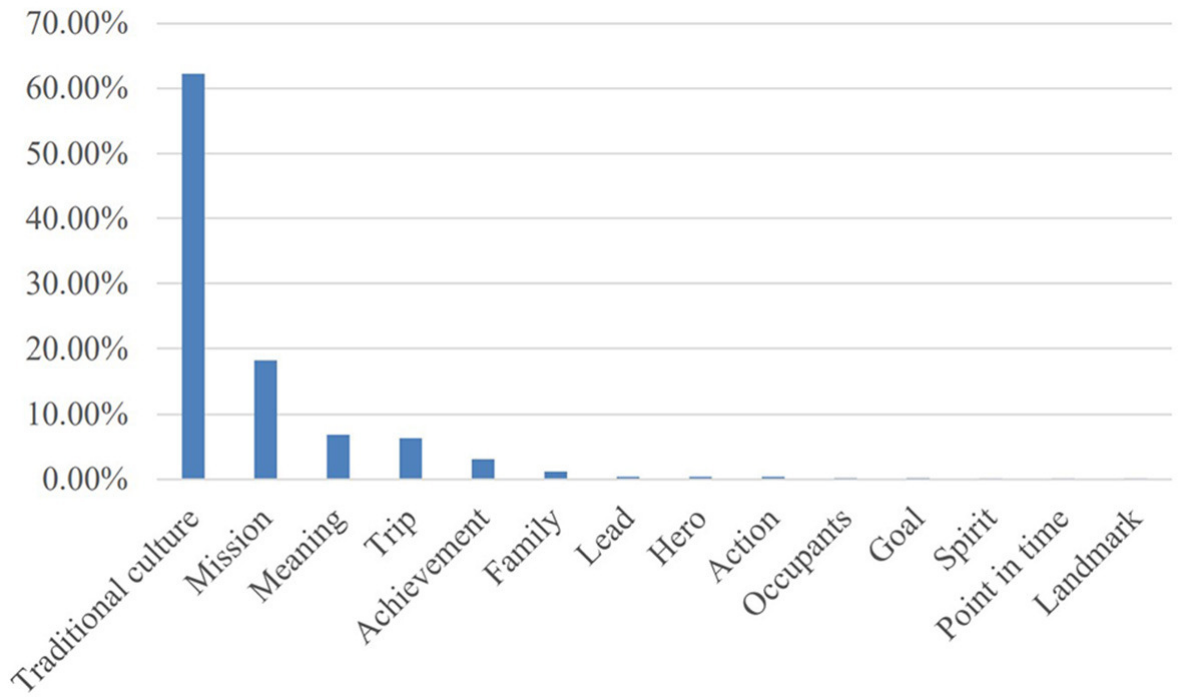
Micro-level semantic domains of conceptual metaphors.

The table and Figures 3, 4 above show that there are mainly seven conceptual domains and 18 conceptual sub-domains in the series of news by the case of Shenzhou XIII aerospace. Then, the characteristics of these conceptual metaphors will be discussed.

Similarly, the “Traditional culture (Shenzhou/Tiangong/Tianhe/Tianzhou/Tianwen/Wentian/Mengtian/ Chang'e/culture)” domain is most widely used metaphor in the series reports. These metaphor words are from Chinese ancient poems and myths, which express that Chinese people dream about exploring space and having a good longing for space.

The “Strivers” domain is second among all the metaphor domains, which is similar to the news reports of Chang'e-5 and shows the Chinese spirit of persistence and enterprise.

The conceptual metaphor “Significance” is also a key semantic domain in these news reports, in which the “Meaning” sub-domain is the most widely used metaphor, and “Achievements (accomplish/accomplishment, achieve/achievement)” and “Action (stride, seize)” are also used to speak highly of Shenzhou XIII, which make a great contribution to Chinese aerospace industry. The achievement of Shenzhou XIII is innovative and extraordinary.

The “Journey” metaphor is also used. It includes “Trip (Long March, Journey, travel, trip, voyage, level, foundation, and closed the gap),” “Occupants (occupants),” and “Landmark (milestone)”. These metaphor words are beautiful, pleasant, and directive and show that Shenzhou XIII is exploratory and tentative.

The “Family (home and backup)” metaphor is also utilized and it plays the same role as mentioned earlier. “Leadership (leading, guidelines, and forerunners)” and “Hero (hero)” metaphors show that the Chinese aerospace industry is the leader in the world and Chinese aerospace workers are wise and brave and make great contributions to the aerospace industry. The “Age (age)” metaphor expresses that the Chinese aerospace industry is at a new stage.

Next, the conceptual metaphor domains can be divided into three categories, and domains in each category and their word frequency are listed as follows:

1. Chinese aerospace industry is going upward (162): Shenzhou XIII is mission (153) > Shenzhou XIII is action (3) = Shenzhou XIII is hero (3) > Shenzhou XIII is goal (2) > Shenzhou XIII is spirit (1).
2. China has a good wish for the space industry (583): Shenzhou XIII is traditional culture (520) > Shenzhou XIII is trip (53) > Shenzhou XIII is family (10).
3. Shenzhou XIII is of great significance (90): Shenzhou XIII is meaning (57) > Shenzhou XIII is achievement (26) > Shenzhou XIII is lead (3) > Shenzhou XIII is occupants (2) > Shenzhou XIII is time (1) = Shenzhou XIII is landmark (1).

To sum up, the conceptual metaphors in the news series of *China Daily* by the case of Shenzhou XIII and Chang'e-5 reflect that Chinese aerospace is going upward, Chang'e-5 is of great significance and China has a good wish in future for the space industry.

## Discussion

This study mainly explores the scope of aerospace news from 2008 to 2022. Compared to the 28 news editorials discussed by Li (2016) from 1986 to 2016, it has an advantage of the corpus and the classification of metaphorical concepts. China's image constructed by this study is closer to the development level of modern China's aerospace industry, which presents significant differences in the constructed image of China by Li's (2016) study. Based on the metaphorical analysis above, it can be seen that *China Daily* takes an important role in constructing the image of the Chinese aerospace industry from several aspects:

(1) Chinese aerospace industry is a dream-building action with lofty goals. In the case of the space probe by the series of *China Daily*, the “Traditional Culture (Chang'e, Yutu/Jade Rabbit, Shenzhou, Tiangong, Tianwen, and Queqiao)” metaphor expresses that Chinese people's yearning for the moon and the spirit of exploration for the outer space. For example:

[1] China has seen breakthroughs in scientific explorations like the **Tianwen-1** (Mars mission), **Chang'e-5** (lunar probe), and **Fendouzhe** (deep-sea manned submersible).
[2] Then the two space labs—**Wentian or “Quest for Heavens”**, and “**Mengtian” or “Dreaming of Heavens”**—will be lifted to complete the **Tiangong** station.

Culture deeply influences people's values, and our values always form a coherent system with the metaphorical concepts we live by, thus, metaphorical expressions embed people's culture (Lakoff and Johnson, 1980; Deignan, 2003). Chinese culture is rooted in the heart of Chinese people, there is no doubt that Chinese culture deeply influences metaphors in *China Daily* news reports. Even in the field of technology, it embodies the confidence of traditional Chinese culture.

(2) Chinese aerospace industry is an enterprising action that represents the prosperity and progress of China. In the case of the space probe by the series of *China Daily*, the “Goal (start, goal, dream, and wake-up call)” and “Spirit (spirit)” metaphors show that China is optimistic and confident about the future of the aerospace industry. For example:

[3] the successful return of the Chang'e-5 probe is a vivid demonstration of the lunar exploration **spirit** of “pursuing dreams, daring to explore, collaborating in tackling difficulties, and win–win cooperation”, which shares a great similarity with the Olympic motto of “faster, higher, and stronger”.
[4] The Chang'e-5 mission will be yet another historic **moment** for China's lunar program.

(3) Chinese aerospace industry is an exploratory action that is constantly forging ahead and pursuing. In the case of space probes by the series of *China Daily*, the “Mission (mission, exploration, attempt, prototype, and endeavor)” metaphor is widely used, which makes up 23.91% and 18.32%, respectively. It constructs the image of Chinese people advancing bravely and being modest. In addition, the “Trip (step, phase, stage, course, and on its way/pave the way)” metaphor highlights that the Chinese aerospace industry is at the developing stage and China still needs to strive and explore continuously. For example:

[5] Pei Zhaoyu, a spokesman for the mission, said if the mission is successful, it will be a milestone in the nation's lunar exploration **endeavors** and will show the world China's scientific, technological, and engineering capabilities.
[6] A next-generation engine, that will **pave the way** for lunar exploration, was successfully tested on Sunday.

The conceptual metaphors reflect the proactive spirit of China's aerospace industry, which is consistent with the speech by Xi (2021) “We should continue to leverage the advantages of the new national system, increase efforts in independent innovation, make further efforts to promote the innovative development of China's aerospace science, space technology, and space applications, actively engage in international cooperation, and make new and greater contributions to enhancing human welfare.”

(4) Chinese aerospace industry is a leading action that opens a new chapter and leads a new journey. The series of reports of a space probe in *China Daily* widely used the “Significance (success/successful/successfully, achieve/achievement, historic, breakthrough/breakthroughs, strategic, lift, and power)” metaphor, which speaks highly of two missions. The “Difficulty (challenge/challenging, complicated, and overcame)” metaphor highlights the challenges of Chang'e-5. “Leadership (heads and lead),” “Chapter (chapter),” and “Music (trilogy and rehearsal)” metaphors emphasize the significance of Chang'e-5, which promotes the development of the Chinese aerospace industry and brings China's aerospace industry to the forefront of the world. For example:

[7] Chang'e-5 will achieve several **breakthroughs**, including automatic sampling, ascending from the moon without a launch site, and completing an unmanned docking 400,000 km above the lunar surface.
[8] Adopting a complicated technological approach, the Chang'e-5 mission **overcame** many technological challenges, including China's first spacecraft liftoff from an extraterrestrial body and the first unmanned rendezvous and docking in lunar orbit.

It is the spirit of Chinese astronauts and scientists to be “particularly capable of enduring hardships, fighting, tackling challenges, and dedicating themselves”, which is reflected in the news reports of Chang'e-5. These spirits greatly promote the development of China's aerospace, and become a great force for the rise of China's aerospace industry.

(5) Chinese aerospace industry is a brave action that dares to be the first to live in space. The “Journey (journey, travel, trip, and occupants)” metaphor used in the news reports of Shenzhou XIII shows that China dares to forge ahead, breaks the Convention, and advances the development of the world's aerospace industry one step. For example:

[9] ……and will become the first **occupants** of the core module after their spacecraft docks with the module, which is **traveling** in a low-Earth orbit hundreds of kilometers above the ground.

(6) Chinese aerospace industry is a great activity to create a community with a shared future for mankind. The “Share (share and willingness)” metaphor in the news reports of Chang'e-5 emphasizes that China is willing to share scientific and technological achievements with other countries and promote the world aerospace industry, which fully reflects the idea of a community with a shared future for mankind. For example:

[10] The Chang'e-5 was the first space expedition from any country to bring back lunar samples in decades, and China has put in place measures to ensure these can be **shared** with the international research community.

In the technological field, China is willing to share the fruit with other countries and promotes common progress. Just as Tang and Zhang (2011) believe that “the news dissemination of major aerospace practice activities can explain China's intention to adhere to independent innovation and maintain a harmonious world of lasting prosperity. It showcases the continuous progress of China's technological field, constructs the image of China actively participating in international exchanges, which gradually changes the impression of China constructed by Western media as politically closed, culturally mysterious, and socially chaotic, and further attracts more countries to accept China's concept of ‘common development' and deepen cooperation”.

## Conclusion

In conclusion, using conceptual metaphors, this article investigates metaphor words in 460 series reports of *China Daily* in the case of space probes, all of which are working together to construct the six types of China's image in the aerospace industry. It found that the conceptual metaphors used in the case of the space probe by the series of *China Daily* construct the image of China in the aerospace industry characterized by the following features: the Chinese aerospace industry is a dream-building action with lofty goals, an enterprising action that represents the prosperity and progress of China, an exploratory action that is constantly forging ahead and pursuing, a leading action that opens a new chapter and leads a new journey, a braving action that dares to be the first to live in the space, and an achieving action to create a community with a shared future for mankind.

According to Lakoff and Johnson (2005, p. 27), “The essence of metaphor is understanding and experiencing one kind of thing in terms of another.” In this process, the metaphorical target is constructed by our understanding and experience, which can reflect people's thoughts, emotions, and cognition about the source. That is to say, “We talk about arguments that way because we conceive of them that way—and we act according to the way we conceive of things (1980).” Although the news reports are about the development of the aerospace industry, they reflect the value orientation and ideology of China. Through examining the conceptual metaphors used in the aerospace news reports, this article investigates China's image constructed by Chinese journalists in the aerospace field, which is a powerful counterattack to the Western construction of China's national image. However, in terms of methodology, the current study mainly adopts the methods of manual reading, identification, and recognition when selecting metaphorical words, so subjectivity and omissions are inevitable. Thus, in future research, the corpus tools can be used to examine the metaphor words and enhance the objectivity of the research.

## Data availability statement

The datasets presented in this study can be found in online repositories. The names of the repository/repositories and accession number(s) can be found in the article/Supplementary material.

## Ethics statement

The studies involving human participants were reviewed and approved by the National University of Defense Technology. The written informed consent to participate in this study was provided by the participants or their legal guardian/next of kin.

## Author contributions

XL and DL contributed to the conception and design of the study. DL organized the database. XL performed the statistical analysis and wrote the first draft of the manuscript. All authors contributed to the manuscript revision and read and approved the submitted version.

## Footnotes

^1^About *China Daily* Group. https://www.chinadaily.com.cn/e/static_e/about (2022-02-09).
