# Supplementary material for: Conceptual metaphors and image construction of China in the space probe reports of China Daily: a social cognitive approach
Source: Front Psychol. 2023 Jun 8;14:1202988. doi: 10.3389/fpsyg.2023.1202988 (PMC10286809; doi:10.3389/fpsyg.2023.1202988)
Supplement: Supplementary file 1 [file Data_Sheet_1.zip › supplementary material/Shenzhou-13 metaphorical sentences.docx]

Shenzhou

1. On June 11, 2013, Wang Yaping went to space on Shenzhou X spaceship with astronauts Nie Haisheng and Zhang Xiaoguang and returned on June 26.

2. She went again on Oct 16, 2021, on Shenzhou XIII spaceship with astronauts Zhai Zhigang and Ye Guangfu.

3. On Nov 7, she became China's first female spacewalker when she took part in the Shenzhou XIII mission's first extravehicular activity with mission commander Major General Zhai Zhigang.

4. TT&C missions of the Shenzhou and Tianzhou spacecraft series, Tianhe core module, Chang'e lunar probe series, and Tianwen-1 Mars probe have been completed successfully.

5. On Nov 7, she became China's first female spacewalker when she took part in the Shenzhou XIII mission's first extravehicular activity with mission commander Major General Zhai Zhigang.

6. China's Shenzhou XIII crew carries out docking test "Screen image taken at Beijing Aerospace Control Center on Jan 8, 2022 shows the Shenzhou XIII astronauts in China's space station core module conducting the manual rendezvous and docking experiment with the Tianzhou 2 cargo craft.

7. China's Shenzhou XIII mission crew conducted a remotely controlled docking test from inside the Tiangong space station on Saturday, according to the China Manned Space Agency.

8. The Shenzhou XIII astronauts in China's space station core module have completed the manual rendezvous and docking experiment with the Tianzhou 2 cargo craft, the China Manned Space Agency (CMSA) said Saturday.

9. The six launches will be used to deploy the Shenzhou XIV and XV mission crews to the Tiangong station, which is circling the Earth in a 400-kilometer-high orbit; to transport the Tianzhou 4 and 5 robotic cargo spaceships to the station for refueling and resupply operations; and to deliver two large space labs to dock with Tiangong, the report said.

10. Mission planners at CASC have said that of the six spacecraft, the first to be launched will be the Tianzhou 4, followed by the Shenzhou XIV manned spacecraft.

11. Then the two space labs－Wentian, or ""Quest for Heavens"", and Mengtian, or ""Dreaming of Heavens""－will be lifted to complete the Tiangong station.

12. The Tianzhou 5 will be the fifth, and the final one will be the Shenzhou XV.

13. Currently, Tiangong is occupied by the Shenzhou XIII mission crew－Major General Zhai Zhigang, Senior Colonel Wang Yaping and Senior Colonel Ye Guangfu－who arrived at the station in mid-October and are scheduled to stay there for six months.

14. On Oct 16, the Shenzhou XIII mission successfully sent the three astronauts into the core module of the Tiangong space station called Tianhe, meaning Harmony of the Heavens.

15. This was the fourth time that the taikonauts conducted EVAs during the construction of the country's space station and the second by the Shenzhou XIII crew.

16. The Shenzhou XIII crew will continue their in-orbit work to welcome the coming new year.

17. China launched the Shenzhou XIII spaceship on Oct 16, sending three taikonauts on a six-month mission to construct its space station.

18. Shenzhou astronauts conduct 2nd spacewalk "China's Shenzhou XIII astronaut Senior Colonel Ye Guangfu begins activities outside the space station core module Tianhe on Sunday.

19. China's Shenzhou XIII mission crew conducted their second extravehicular activity on Sunday.

20. The Shenzhou XIII mission was launched on Oct 16 by a Long March 2F carrier rocket that blasted off from the Jiuquan Satellite Launch Center in northwestern China's Gobi Desert, with the crew soon entering the Tiangong space station.

21. Shenzhou XIII is the fourth spacecraft to visit the Tiangong station and the second crewed ship to transport astronauts to the orbiting outpost.

22. China's Shenzhou XIII crew starts second spacewalk "China's Shenzhou XIII mission crew started their second extravehicular activity, or spacewalk, on Sunday evening, according to the China Manned Space Agency.

23. Shenzhou XIII crew to take second spacewalk "

24. China's Shenzhou XIII mission crew is scheduled to carry out their second extravehicular activity, or spacewalk, on Sunday, according to the China Manned Space Agency.

25.As in the case of students' attendance at the livestreamed experimental demonstrations given earlier by the three Chinese taikonauts, as they are often addressed on the mainland, on board China's spaceship Shenzhou XIII, direct interactions with the motherland's athletic experts and practitioners surely bring to the Hong Kong and Macao SARs enviable gains in specialized knowledge and horizon extension, this time over personal and community development in popular and elitist sports.

26. Eight years ago, the crew of Shenzhou X spaceship gave the first lecture from space to Chinese students.

27. Pupils in Tibet get a lesson from space station "Students in Lhasa, Tibet autonomous region, attend a live class given by Shenzhou XIII crew members on Thursday.

28. Major General Zhai Zhigang, Senior Colonel Wang Yaping and Senior Colonel Ye Guangfu, all members of the Shenzhou XIII mission crew, greeted students, teachers and other participants when the lecture started at 3:54 pm.

29. Chinese astronauts' lecture from space draws millions of viewers "Photo taken in South China's Macao on Dec 9, 2021 shows Shenzhou XIII crew member Wang Yaping giving a special lecture at China's space station via video call.

30. Watch it again: Space class from Tiangong station "Crew members of China's Shenzhou XIII mission are scheduled to give a space-based lecture on Thursday afternoon from the orbiting Tiangong space station to students around the world.

31. First crew of space station recovering well "Major General Nie Haisheng (center), Major General Liu Boming (right) and Senior Colonel Tang Hongbo from the Shenzhou XII space mission meet the media at Beijing Aerospace City on Dec 7, 2021.

32. Shenzhou XII astronauts now under observation ahead of return to training

33. The three astronauts involved in the Shenzhou XII mission have recovered well after their 3-month spaceflight and will return to regular training once related health assessments are completed.

34. The three Shenzhou XII astronauts－Major General Nie Haisheng, Major General Liu Boming and Senior Colonel Tang Hongbo－have completed the convalescence phase.

35. The Shenzhou XII crew, commanded by Nie, spent 92 days in space after the spacecraft was launched on June 17 on a Long March 2F carrier rocket, setting a new national record for the longest human spaceflight.

36. Crew members of China's Shenzhou XIII mission are scheduled to give a space-based lecture on Thursday afternoon from the orbiting Tiangong space station to students around the world, the China Manned Space Agency said on Monday.

37. During the Shenzhou X mission in June 2013, she carried out the nation's first space-based lecture inside an experimental space station module to more than 60 million Chinese students."

38. Center in northwestern China's Gobi Desert, with the crew soon entering the Tiangong station.

39. In June 2013, she took part in the Shenzhou X mission that lasted nearly 15 days.

40. The three astronauts of China's Shenzhou XIII mission sent their greetings to young volunteers in a video sent from the orbiting Tiangong space station.

41. The statement issued by the agency on Thursday did not mention which member of the crew－Major General Zhai Zhigang, Senior Colonel Wang Yaping and Senior Colonel Ye Guangfu－will host the lecture, but Lin Xiqiang, the agency's deputy director, told a news conference in mid-October, right before Shenzhou XIII's launch, that ""Teacher Wang will soon bring her second space lecture to you.""

42. In June 2013, Wang took part in the Shenzhou X mission that lasted nearly 15 days.

43, Early last month, Wang Yaping, 41, became China's first female spacewalker when she took part in the Shenzhou XIII mission's first extravehicular activity."

44. The Shenzhou XIII mission launched on Oct 16 on a Long March 2F carrier rocket that blasted off from the Jiuquan Satellite Launch Center in northwestern China's Gobi Desert, with the crew entering Tiangong later that day.

45. The trip are scheduled to spend six months working in the station, making it China's longest manned space mission."

46. The Shenzhou XIII crew, with Major General Zhai Zhigang and Senior Colonel Ye Guangfu acting as photographers, took pictures of Senior Colonel Wang Yaping inside the core module ""Tianhe"".

47. A video released on Tuesday shows Zhai Zhigang, a crew member of Shenzhou XIII, practicing tai chi on the Tiangong-1 space lab module with flowing and graceful movements.

48. In fact, the tradition of Chinese astronauts practicing tai chi started in 2012 when the country's Shenzhou IX crew member Liu Yang first did so in space and called the experience comfortable.

49. Before returning from the Shenzhou XII mission in September, taikonaut Nie Haisheng also practiced tai chi on board the Tianhe core cabin during an Earth-space video call with Hong Kong youths.

50. On Sina Weibo and WeChat Moments, major microblogging service platforms in China, posts about Deng, a 55-year-old member of the People's Liberation Army Astronaut Division, have surged since mid-October when China launched its latest manned flight－Shenzhou XIII－to its Tiangong space station.

51. That first group included Yang Liwei, the first Chinese citizen sent into space in 2003, Zhai Zhigang, who conducted his second spacewalk on Sunday, and Nie Haisheng, who returned from the Shenzhou XII mission in September.

52. He was selected for the backup crew for the Shenzhou IX, X and XI missions, but has yet to make it into space.

53. In June 2013, both the father and daughter were at the Jiuquan Satellite Launch Center in northwestern China, preparing for the Shenzhou X mission.

54. Astronaut in orbit takes historic step "Senior Colonel Wang Yaping, 41, became China's first female spacewalker on Sunday evening when she took part in the Shenzhou XIII mission's first extravehicular activity with mission commander Major General Zhai Zhigang.

55. It said the Shenzhou XIII crew will carry out one to two more spacewalks in the coming months.

56. In March 2012, she was part of the backup crew for the Shenzhou IX mission, and in June 2013, she took part in the Shenzhou X mission, which lasted nearly 15 days.

57. During the Shenzhou X flight, Wang gave China's first space-based lecture inside the Tiangong I experimental module to more than 60 million Chinese students at about 80,000 schools across the country.

58. She was selected for Shenzhou XIII in December 2019 and was also a member of the backup crew for the Shenzhou XII mission."

59. Shenzhou XIII astronauts make history in 1st spacewalk "Screen image taken at Beijing Aerospace Control Center on Nov. 8, 2021 shows Chinese taikonaut Wang Yaping (R) completing extravehicular activities (EVAs).

60. The Shenzhou XIII mission crew completed their first extravehicular activity, or spacewalk, early on Monday morning, with Senior Colonel Wang Yaping, 41, becoming China’s first female spacewalker.

61. The Shenzhou XIII mission was launched on Oct 16 by a Long March 2F carrier rocket that blasted off from the Jiuquan Satellite Launch Center in northwestern China's Gobi Desert, with the crew soon entering the Tiangong space station, whose name means Heavenly Palace.

62. The Shenzhou XIII crew is tasked with a wide range of assignments, such as performing two to three spacewalks to install a small robotic arm onto a larger one, verifying key procedures and technologies like manual control of the robotic arms and robotic arm-assisted movement of station modules, checking the performance and capability of devices inside the station, and testing support instruments for astronauts' life and work in long-term flights.

63. The Shenzhou XIII mission crew has carried out an emergency evacuation exercise recently, China Central Television reported on Sunday.

64. The three-member crew — Major General Zhai Zhigang, Senior Colonel Wang Yaping and Senior Colonel Ye Guangfu — practiced evacuating the Tiangong space station and returning to their Shenzhou XIII spacecraft in a scenario simulating the station losing its inner pressure after being hit by space debris.

65. Their peers in the Shenzhou XII mission, which lasted three months and concluded in mid-September, performed two spacewalks."

66. Chinese astronauts undergo recuperation after quarantine "A see-off ceremony for three Chinese astronauts of the Shenzhou XII manned space mission is held at the Jiuquan Satellite Launch Center in Northwest China, June 17, 2021.

67. The crew members of China's Shenzhou XII spaceflight have finished their quarantine and are in the recuperation period, the China Manned Space Agency said on Saturday.

68. The Shenzhou XII spacecraft was launched on a Long March 2F carrier rocket that blasted off on June 17 from the Jiuquan Satellite Launch Center in northwestern China.

69. The astronauts entered Tiangong later that day after the two spacecraft docked with each other, becoming the first inhabitants of the station.

70. Currently, the Tiangong station is hosting the Shenzhou XIII crew -- Major General Zhai Zhigang, Senior Colonel Wang Yaping and Senior Colonel Ye Guangfu – who were lifted in mid-October atop a Long March 2F at the Jiuquan center.

71. Shenzhou XIII crew ready for first spacewalk "The Shenzhou XIII mission crew will soon carry out their first extravehicular activity, or spacewalk, according to the China Manned Space Agency.

72. The mother of a 5-year-old girl, Wang is China's second female astronaut to take part in a spaceflight – she took part in the Shenzhou X mission in June 2013.

73. In the Shenzhou XIII flight, she will become the first Chinese woman to enter a space station and also the first to carry out a spacewalk."

74. Shenzhou XII CPC flag put on display at museum "The Communist Party of China flag carried by the country's Shenzhou XII manned spaceflight is delivered to the Museum of the CPC in a ceremony on Nov 4, 2021.

75. The Communist Party of China flag carried by the country's Shenzhou XII manned spaceflight has become a permanent exhibit at the Party's museum in Beijing, according to China Central Television.

76. The flag was carried by the Shenzhou XII mission crew – Major General Nie Haisheng, Major General Liu Boming and Senior Colonel Tang Hongbo – during their 92-day space journey, so far the longest flight by Chinese astronauts.

77. It was placed on the wall inside China's Tiangong space station during the mission.

78. The Shenzhou XII spacecraft was launched on a Long March 2F carrier rocket that blasted off on June 17 from the Jiuquan Satellite Launch Center in northwestern China.

79. Currently, the Tiangong station is hosting the Shenzhou XIII crew — Major General Zhai Zhigang, Senior Colonel Wang Yaping and Senior Colonel Ye Guangfu – who traveled there in mid-October atop a Long March 2F at the Jiuquan center.

80. China's space progress benefits us all "Screen image captured at Beijing Aerospace Control Center in Beijing, capital of China, Oct 16, 2021 shows China's Shenzhou XIII crewed spaceship having successfully docked with the radial port of the space station core module Tianhe.

81. For instance, China has closed the gap with the US in space science and the exploration of outer space.

82. On Saturday three Chinese astronauts entered China's space station core module Tianhe, hours after the Shenzhou XIII spaceship was successfully launched on Saturday.

83. Crew on board the Shenzhou XIII spacecraft - Zhai Zhigang (C), Wang Yaping (R) and Ye Guangfu (L).

84. Test conducted to verify spacecraft technology, FM says "The Shenzhou XIII spacecraft and the Long March 2F carrier rocket are moved from a testing facility to a launch tower at the Jiuquan Satellite Launch Center on Thursday.

85. Wang was selected for the Shenzhou XIII mission in December 2019.

86. In June 2013, Wang took part in the Shenzhou X mission, which lasted nearly 15 days, and gave China's first space-based lecture to Chinese students from inside the Tiangong I experimental module.

87. Netizens' worries answered over female astronauts' health "Screen image captured at Beijing Aerospace Control Center in Beijing, capital of China, Oct 16, 2021, shows the Shenzhou XIII mission crew at the Tiangong space station, embarking on their six-month journey inside the station in the country's longest spaceflight.

88. Astronauts prepare for 6-month module "Screen image captured at Beijing Aerospace Control Center in Beijing, Oct 17, 2021 shows the Shenzhou-13 crew entering the Tianzhou-3 cargo craft.

89. The Shenzhou-13 crew has entered the Tianzhou-3 cargo craft to transfer the cargo, according to the China Manned Space Agency (CMSA) on Sunday.

90. After successfully stationing in China's space station core module Tianhe, the Shenzhou-13 crew opsojournened the hatch of the Tianzhou-3 cargo craft and entered it at 9:50 am (Beijing Time), the CMSA said.

91. Shenzhou XIII mission crew members have started to transfer supplies from the Tianzhou 2 and 3 robotic spaceships to the Tiangong space station, in preparation for their six-month stay inside the orbiting core module.

92. The Shenzhou XIII spacecraft was lifted into space by a Long March 2F carrier rocket early on Saturday morning from the Jiuquan Satellite Launch Center in northwestern China's Gobi Desert.

93. China's longest-yet crewed space mission impressive, expert says "Screen image captured at Beijing Aerospace Control Center in Beijing, capital of China, Oct. 16, 2021 shows China's Shenzhou-13 crewed spaceship having successfully docked with the radial port of the space station core module Tianhe.

94. China's Shenzhou XIII crewed spaceship successfully docked with the port of the space station core module Tianhe on Saturday, a move overseas experts have called another ""key step"" forward in China's space exploration.

95. Three Chinese astronauts aboard the Shenzhou XIII will stay in orbit for six months, making China's longest yet crewed mission for space station construction.

96. Pui Jeng Leong, a media veteran in Brunei, told Xinhua that the successive successful launches of Shenzhou XII and XIII within a four-month period symbolized that China's aerospace industry has entered a new age with Chinese astronauts' long-term stay in a space station, which once again demonstrates that China's aerospace technology has reached a leading level.

97. Crew to stay inside space station for six months, making it China's longest crewed spaceflight "The Shenzhou XIII mission is launched at 12:23 am on Oct 16, 2021

98. The Shenzhou XIII mission crew, including mission commander Zhai Zhigang, Wang Yaping and Ye Guangfu

99. The mission sees first spacewalk by a Chinese woman "The Shenzhou XIII mission is launched at 12:23 am on Oct 16, 2021

100. The Shenzhou XIII mission crew, including mission commander Zhai Zhigang, Wang Yaping and Ye Guangfu.

1. The crew enter China's Tiangong space station at 9:58 am on Oct 16, 2021, embarking on their six-month journey inside the station in the country's longest crewed spaceflight.

2. 4th spacecraft to visit Tiangong station and 2nd crewed ship to transport astronauts to the station "The Shenzhou XIII mission is launched at 12:23 am on Oct 16, 2021

3. Instruments, ink stones, and gifts…Astronauts make space station new home

4. Media and public attention has shifted to Chinese astronauts' life in space after spacecraft Shenzhou XIII, carrying the trio - Zhai Zhigang, Wang Yaping and Ye Guangfu, was successfully launched on Saturday morning.

5. Astronauts onboard the Shenzhou-XIII spaceship have entered the country's space station core module Tianhe to start a six-month journey, according to the China Manned Space Agency.

6. Their Shenzhou XIII spacecraft was lifted by a Long March 2F carrier rocket that blasted off at 12:23 am at the Jiuquan Satellite Launch Center in northwestern China's Gobi Desert.

7. Space station moves closer to generating common human good: "China's Shenzhou XIII crewed spacecraft docks with the Tiangong space station on Saturday morning, with astronauts ready to enter the station to start a six-month journey.

8. The successful launch of the Shenzhou XIII manned spacecraft from Jiuquan, Gansu province, in the early hours of Saturday marked a big stride toward the conclusion of trials for key technologies for China's first space station Tiangong.

9. With three astronauts on board, Shenzhou XIII will dock with Tianhe core module about eight hours later.

10. Shenzhou XIII is China's sixth manned mission in Tianhe's technology trials, but its importance should not be underestimated, as it will evaluate the functions and performances of all the systems and work units of the space station, and help complete its construction.

11. According to the China Manned Space Agency, if all goes well, the country will launch six other missions－Tianzhou 4 cargo spacecraft and Shenzhou XIV manned spacecraft, Wentian and Mengtian experimental spacecraft, Tianzhou 5 cargo spacecraft, and Shenzhou XV manned spacecraft－during the construction stage of the space station which is scheduled for completion before the end of 2022.

12. China's spacecraft docks with space station, astronauts enter core module "Screen image captured at Beijing Aerospace Control Center in Beijing, capital of China, Oct 16, 2021 shows China's Shenzhou-13 crewed spaceship docking with the radial port of the space station core module Tianhe.

13. China's Shenzhou-13 crewed spaceship successfully docked with the radial port of the space station core module Tianhe on Saturday, according to the China Manned Space Agency.

14. The three Chinese astronauts onboard the Shenzhou-13 spaceship entered the country's space station core module Tianhe on Saturday, according to the China Manned Space Agency.

s

15. Shenzhou XIII crew starts epic mission "China launched the Shenzhou XIII mission to the Tiangong space station early on Saturday morning, marking the start of the country's longest spaceflight.

16. Carrying the Shenzhou XIII spacecraft, a 20-story-tall Long March 2F carrier rocket blasted off at 12:23 am at the Jiuquan Satellite Launch Center in northwestern China's Gobi Desert, brightening the dark sky as it roared up from a huge service tower.

17. The mother of a 5-year-old girl, Wang is China's second female astronaut to take part in a spaceflight. She took part in the Shenzhou X mission in June 2013.

18. The mission is expected to become the longest crewed spaceflight by China, doubling the time spent in the Shenzhou XII mission.

19. The crewed spaceship Shenzhou XIII, atop a Long March-2F carrier rocket, is launched from the Jiuquan Satellite Launch Center in Northwest China's Gobi Desert, Oct 16, 2021.

20. Pang Zhihao, a spaceflight researcher in Beijing and a former analyst at the China Academy of Space Technology, said that the Shenzhou XIII mission will lay a solid foundation for the next steps in the Tiangong space station program.

21. It will also check whether the core module and the Shenzhou spaceship can withstand a tough environment during a long-term flight,"" he said.

22. With three astronauts on board, Shenzhou XIII will dock with Tianhe core module about eight hours later.

23. Shenzhou XIII crew set out for launch tower "The crewed spaceship Shenzhou-13, atop a Long March-2F carrier rocket, is launched from the Jiuquan Satellite Launch Center in Northwest China's Gobi Desert, Oct 16, 2021.

24. China's Shenzhou-13 crewed spaceship docks with space station module

25. China's Shenzhou-13 crewed spaceship successfully docked with the radial port of the space station core module Tianhe on Saturday, according to the China Manned Space Agency."

26. Yantai astronaut selected for Shenzhou XIII "Senior Colonel Wang Yaping, 41, a native of Shandong province, joined the PLA Air Force in August 1997 and served as deputy squadron commander before joining the second group of the PLA Astronaut Division in May 2010.

27. In March 2012, she was part of the backup crew for the Shenzhou IX mission and in June 2013, she took part in the Shenzhou X mission, which lasted nearly 15 days.

28. Wang was selected for Shenzhou XIII in December 2019."

29. Astronauts to celebrate Lunar New Year in space "Crew on board the Shenzhou XIII spacecraft - Zhai Zhigang (C), Wang Yaping (R) and Ye Guangfu (L).

30. Crew members of the upcoming Shenzhou XIII mission will become the first Chinese to spend the nation's most important festival－Spring Festival, or Chinese Lunar New Year－in outer space.

31. Wang took part in the Shenzhou X mission, which lasted nearly 15 days, in June 2013.

32. During the mission, she gave a 40-minute space lecture from inside the Tiangong I experimental module.

33. Launch of Shenzhou XIII scheduled for Saturday "The crew of China's Shenzhou XIII space mission－astronauts Zhai Zhigang (center), Wang Yaping (right) and Ye Guangfu－meet the media at the Jiuquan Satellite Launch Center on Thursday at a news conference on the mission.

34. China plans to launch the Shenzhou XIII manned mission early on Saturday morning, sending three astronauts to spend six months inside the Tiangong space station.

35. It is expected to become the longest space journey by Chinese astronauts, doubling the time their peers spent on the Shenzhou XII mission.

36. The Shenzhou XIII spacecraft is scheduled to be launched at 12:23 am on Saturday by a Long March 2F carrier rocket at the Jiuquan center to take mission commander Major General Zhai Zhigang, Senior Colonel Wang Yaping and Senior Colonel Ye Guangfu to Tiangong's core module, Tianhe, or Harmony of Heavens, Lin said at the news conference.

37. Wang became the second female astronaut to take part in a spaceflight as a crew member of the Shenzhou X mission in June 2013.

38. Liu Yang, who took part in the Shenzhou IX mission in June 2012, was China's first woman in space.

39. Zhai is the first Chinese astronaut to conduct a spacewalk, a feat he achieved in September 2008 during the Shenzhou VII mission, which he commanded.

40. Huang Weifen, the chief trainer of Chinese astronauts, said that to help the Shenzhou XIII crew to better prepare for the coming flight and familiarize themselves with the station as quickly as possible, mission planners arranged video and face-to-face meetings between them and the Shenzhou XII astronauts, who shared their experiences of living and working inside the Tiangong.

41. Shenzhou XIII will be the fourth spacecraft to visit the Tiangong station and the second crewed ship to transport astronauts to the orbiting outpost.

42. The spacecraft stack is in good condition and is ready for rendezvous and docking with Shenzhou XIII, Lin said.

43. Shenzhou XIII will be the last mission in the technological verification phase of the Tiangong space station program.

44. The crew members selected for Shenzhou XIII "Major General Zhai Zhigang, 55, a native of Heilongjiang province, joined the People's Liberation Army Air Force in June 1985.

45. Zhai was one of two alternates for Yang Liwei, who made China's first manned spaceflight, on the Shenzhou V mission in October 2003, and was part of the backup crew for the Shenzhou VI mission in October 2005.

46. In September 2008, he made his first space mission on board Shenzhou VII.

47. Launch of Shenzhou XIII mission scheduled for Saturday "China plans to launch the Shenzhou XIII manned mission early on Saturday morning, sending three astronauts to spend six months inside the Tiangong space station.

48. Last month's safe return of the three crew members of the Shenzhou XII mission was an achievement not just for China's space program but also for Donghua University in Shanghai.

49. The three astronauts selected for Shenzhou XIII "Zhai Zhigang

50. In September 2008, he undertook his first space mission onboard Shenzhou VII. He was accompanied by Liu Boming and Jing Haipeng.

51. Zhai was selected for Shenzhou XIII in December 2019."

52. Shenzhou XIII space mission to begin on Saturday "Crew on board the Shenzhou XIII spacecraft - Zhai Zhigang (C), Wang Yaping (R) and Ye Guangfu (L).

53. China plans to launch the Shenzhou XIII manned spacecraft early on Saturday morning, carrying three astronauts who will stay inside the country's Tiangong space station for six months, one of the lead officials in the country's manned space program said.

54. Second space station crew to launch soon "The Shenzhou XIII spacecraft and the Long March 2F carrier rocket are moved from a testing facility to a launch tower at the Jiuquan Satellite Launch Center on Thursday.

55. China will soon conduct its Shenzhou XIII manned space flight, sending three astronauts to stay in the country's Tiangong space station for six months.

56. The Long March 2F carrier rocket that will launch the Shenzhou XIII spacecraft this month was moved to a launch tower at the Jiuquan Satellite Launch Center in northwestern China's Gobi Desert on Thursday morning.

57. During the Shenzhou XIII mission, the astronauts, whose names have yet to be disclosed, will be mainly tasked with demonstrating and testing key technologies involved in the assembly and future operations of the Chinese space station, according to mission planners at the agency.

58. Like their peers in the Shenzhou XII mission, they will carry out spacewalks to test the robotic arm and other instruments for extravehicular activities, and will also conduct scientific experiments to accumulate experience for the station's construction and operations, planners said.

59. Shenzhou XIII will be the fourth spacecraft to visit China's permanent space station, named Tiangong, or Heavenly Palace, and the second crewed ship to transport astronauts to the orbiting outpost.

60. The first astronauts to stay in Tiangong－Major General Nie Haisheng, Major General Liu Boming and Senior Colonel Tang Hongbo－finished their 92-day Shenzhou XII mission in mid-September.

61. The crew of Shenzhou XIII will enter China's space station and stay there for six months, after a three-month mission of the Shenzhou XII crew.

62. On Sept 3, the three Shenzhou-12 crew members had a live conversation with about 300 representatives of university and middle school students, teachers, and sci-tech researchers in Hong Kong SAR.

63. Another video showing Tang spinning a pen in Shenzhou XII has been played nearly 1.6 million times.

64. China prepares to launch Shenzhou XIII manned spaceship "The combination of the Shenzhou XIII manned spaceship and a Long March 2F carrier rocket is seen being transferred to the launch site, on Oct 7, 2021.

65. The combination of the Shenzhou XIII manned spaceship and a Long March 2F carrier rocket has been transferred to the launching area, and the spaceship will be launched in the near future at an appropriate time, the China Manned Space Agency (CMSA) said Thursday.

66. China plans to conduct its Shenzhou XIII manned space flight this month, sending three astronauts to stay six months inside the country's Tiangong space station.

67. The first astronauts inside Tiangong－Major General Nie Haisheng, Major General Liu Boming and Senior Colonel Tang Hongbo－finished their 92-day Shenzhou XII mission in mid-September.

68. Zhao Lijian, a spokesman for the Foreign Ministry, said at a daily briefing after the Shenzhou XII crew returned to Earth that China's manned space programs have contributed greatly to mankind's peaceful development of outer space, and the country will continue broadening and deepening its cooperation and communication with the international community to build the Chinese space station into a shared laboratory to bring benefits to all people on the globe.

69. On Oct 15, 2003, the country carried out its first manned space flight, sending Yang Liwei on a 21-hour journey around the mother planet in the Shenzhou V spacecraft.

70. In October, a second group of three astronauts is scheduled to be sent to Tianhe in the Shenzhou XIII crewed spacecraft.

71. The combination of the station's core module and the cargo spaceship had been in good condition, the agency said, noting the orbiting pair will soon welcome the Tianzhou 3 cargo ship and the Shenzhou XIII crewed spacecraft that will carry three astronauts.

72. Shenzhou XII crew returns to Earth "Astronauts Nie Haisheng (center), Liu Boming (right) and Tang Hongbo wave after they landed in the reentry capsule of the Shenzhou XII mission in Inner Mongolia autonomous region on Friday.

73. Crew members of the Shenzhou XII mission returned to Earth on Friday afternoon, concluding a historic journey in China's Tiangong space station.

74. Nie and his crew spent 92 days in space since their Shenzhou XII spacecraft was launched at the Jiuquan center on June 17.

75. On Thursday morning, they completed their final tasks－configuring the space station, transmitting some experimental data back to ground control and arranging materials inside the station－and then left the station and returned to the Shenzhou XII spacecraft, which later departed from the station.

76. Shenzhou XII was launched on a Long March 2F carrier rocket that blasted off on June 17 from the Jiuquan Satellite Launch Center in northwestern China.

77. The three-month Shenzhou XII mission, the nation's seventh manned space mission, is part of the Tiangong program, which aims to complete a three-component station in low-Earth orbit before the end of 2022.

78. The next crewed spaceflight－Shenzhou XIII－is scheduled to be launched in October at the Jiuquan center, sending another three astronauts to the Tiangong station to work there for six months.

79. Shenzhou XII astronauts make triumphant return "Astronauts Nie Haisheng (center), Liu Boming (right) and Tang Hongbo wave after they landed in the reentry capsule of the Shenzhou XII mission in Inner Mongolia autonomous region on Friday.

80. Crew members of the Shenzhou XII mission have returned to the Astronaut Center of China in Beijing's northwestern suburbs after the jetliner carrying them from the Inner Mongolia autonomous region to the capital landed at a military airport on Friday evening.

81. The next crewed spaceflight — Shenzhou XIII — is scheduled to be launched in October at the Jiuquan center, sending another three astronauts to the Tiangong station to work there for six months.

82. Space station crew lands in Inner Mongolia "The reentry capsule carrying crew members of the Shenzhou XII mission has landed in the Inner Mongolia autonomous region.

83. Nie and his crew spent 92 days in space since their Shenzhou XII spacecraft was launched at the Jiuquan center on June 17.

84. On Thursday morning, they completed their final tasks -- configuring the space station, transmitting some experimental data back to ground control and arranging materials inside the station – and then left the station and returned to the Shenzhou XII spacecraft, which later departed the station.

85. The three-month-long Shenzhou XII mission, the nation's seventh manned space trek, is part of the Tiangong program, which aims to complete a three-component station in low-Earth orbit before the end of 2022.

86. The Shenzhou XII crew is scheduled to remain in space until mid-September.

87. It is now connected with the Tianzhou 2 robotic cargo spaceship, launched in late May, and the Shenzhou XII spacecraft, which transported three astronauts to the module in mid-June.

88. Based on project plans, the Tianzhou 3 cargo ship will be launched in September to dock with Tianhe, and in October, another three-member crew will fly in Shenzhou XIII to the module to stay there for six months.

89. As of Friday morning, the Shenzhou XII crew had flown with Tianhe, or Harmony of Heavens－the first and central component of the Chinese space station－for nearly 64 days.

90. Meanwhile, the Shenzhou XIII spacecraft and its rocket are undergoing technical checks at the Jiuquan Satellite Launch Center in northwestern China for the upcoming Shenzhou XIII crewed mission, which is scheduled to transport three astronauts to Tiangong in October, the agency noted.

91. Materials and fuel carried by Tianzhou 3 will support Tiangong's operations and resupply the astronauts on the ongoing Shenzhou XII mission, as well as supply their peers on Shenzhou XIII.

92. Both Tianzhou 3 and Shenzhou XIII are part of the Tiangong program, which aims to complete construction of a three-component space station in low-Earth orbit before the end of 2022.

93. After the Shenzhou XII spacecraft successfully completed a fast automated rendezvous and docking with the orbiting Tianhe module, the crew entered the orbital capsule.

94. Major General Nie Haisheng, Major General Liu Boming and Senior Colonel Tang Hongbo floated into the core module, named Tianhe, or Harmony of Heavens, at 6:48 pm on Thursday, after their Shenzhou XII spaceship linked with the module in a low-Earth orbit about 390 kilometers above the Earth at 3:54 pm.

95. Currently, Tianzhou 2 contains living materials and mission payloads for the Shenzhou XII crew to use, as well as propellants that will be used to refuel the core module.

96. A 20-story-tall Long March 2F carrier rocket blasted off at 9:22 am at the Jiuquan Satellite Launch Center in northwestern China's Gobi Desert, hurtling into the blue sky with the Shenzhou XII spacecraft on top of the gigantic rocket.

97. Crew to head for space station module "Astronauts (left to right) Tang Hongbo, Nie Haisheng and Liu Boming－crew of the Shenzhou XII spacecraft－attend a news conference at the Jiuquan Satellite Launch Center on Wednesday.

98. Ji Qiming, assistant director of the China Manned Space Agency, told reporters at a news conference on Wednesday at the Jiuquan Satellite Launch Center in northwestern China that the crew on board the Shenzhou XII spacecraft－Nie Haisheng, Liu Boming and Tang Hongbo－will be taken into space by a Long March 2F carrier rocket at 9:22 am on Thursday at the launch complex and will become the first occupants of the core module after their spacecraft docks with the module, which is traveling in a low-Earth orbit hundreds of kilometers above the ground.

99. Ji said that after Nie's crew finishes its three-month mission, it will return to Shenzhou XII and then depart from Tianhe and return to Earth to land at the Dongfeng Landing Site in the Inner Mongolia autonomous region.

100. Hao Chun, director of the China Manned Space Agency, told China Daily in April that the Tianzhou 3 cargo ship will be launched from Wenchang in September to dock with Tianhe.

1. The next month, another three-astronaut team will fly to the core module on Shenzhou XIII to work there for six months.

2. Astronauts (from left) Tang Hongbo, Nie Haisheng and Liu Boming, the crew of the Shenzhou XII spacecraft, meet with reporters at the Jiuquan Satellite Launch Center on Wednesday.

3. China to send 3 astronauts to space station "Crew on board the Shenzhou XII spacecraft – Tang Hongbo, Nie Haisheng and Liu Boming (from left to right).

4. Ji said that during their three-month stay inside the module, named Tianhe, or Harmony of Heavens, the all-male crew is tasked with testing and verifying plans, technologies and equipment for crucial elements in space station's construction and operation, such as astronauts' long-term mission arrangements, life-support system, in-orbit resupply, extravehicular activity as well as spacecraft maintenance and repair.

5. Spacewalks planned for Shenzhou missions "Tianzhou 2, a robotic cargo ship, blasts off on a Long March 7 carrier rocket at the Wenchang Space Launch Centerin in Hainan, on May 29, 2021.

6. Astronauts on the upcoming Shenzhou XII mission will engage in spacewalks outside the Tianhe core module of China's Tiangong space station, a key figure in the nation's manned space endeavor said.

7. The astronauts will be launched in June with the Shenzhou XII spacecraft from the Jiuquan Satellite Launch Center in northwestern China to dock with the currently unoccupied Tianhe module.

8. Crew members of the Shenzhou XII and the next three manned spaceflights－Shenzhou XIII, XIV and XV－were selected from those in the first and second groups of astronauts, Yang said.

9. He added that the Shenzhou XII crew is all male, but there will be a woman in each of the next three missions.

10. Shenzhou XIV and XV are expected to take place in 2022, with each crew team staying in space for half a year.

11. Currently, only astronauts in the Shenzhou VII mission, launched in September 2008, made spacewalks."

22. When astronauts from the Shenzhou XII spacecraft board Tianhe in the near future, they will gradually move living materials and mission payloads from Tianzhou 2 to the core module.

23. Hao Chun, director of the manned space agency, said previously that the Shenzhou XII spacecraft, carrying three astronauts, will launch and dock with Tianhe early in June.

24. Hao Chun, director of the manned space agency, said previously that the Shenzhou XII spacecraft, carrying three astronauts, will launch and dock with Tianhe early in June.

25. In October, another three-astronaut team will fly to the core module on Shenzhou XIII to stay there for six months.

26. The Shenzhou XII spacecraft, carrying three astronauts, will follow the robotic cargo ship to dock with the core module in early June.

27. The official said that in September, the Tianzhou 3 cargo ship will be lifted to dock with the core module and the next month, another three-crew team will fly with the Shenzhou XIII to the module to stay there for six months.

28. Yang Liwei, China's first astronaut, smiles to workers after the Shenzhou V manned spaceship successfully landed on the designated site in the Inner Mongolia autonomous region on Oct 16, 2003.

29. Hao Chun, director of the China Manned Space Agency, said earlier this month that the astronauts on board the Shenzhou XII spacecraft will become the first occupants of the core module and will be tasked with undertaking preparation work for the next steps in the station's construction.

30. Before the Shenzhou XII mission, the Tianzhou 2 robotic cargo spacecraft is scheduled to be launched in late May to dock with the currently unmanned core module and then perform autonomous refueling and resupply operations, according to Hao.

31. The official said that in September, the Tianzhou 3 cargo ship will be lifted to dock with the core module and the next month, another three-crew team will fly with the Shenzhou XIII to the module to stay there for six months.

32. After the core capsule was launched, astronauts on the Shenzhou XII and XIII missions and two cargo ships will be launched within a few months to prepare the module for docking with other parts of the station.

33. The Tianzhou 2 cargo ship was transported to the Wenchang facility in mid-April, and the Shenzhou XII manned spacecraft arrived in the Jiuquan Satellite Launch Center in northwestern China at the same time.

34. By comparison, the usable space in the Shenzhou-series crewed spaceship is about 7 cubic meters.

35. After the capsule is launched, astronauts on the Shenzhou XII and XIII missions and two cargo ships will be launched within a few months to prepare the module for docking with other parts of the station.

36. China's next crewed spaceflight will take place soon as the Shenzhou XII spacecraft and the carrier rocket that will lift it have arrived at the Jiuquan Satellite Launch Center in northwestern China.

37. The astronauts for the Shenzhou XII mission are participating in intensive training, it said.

38. Crew members of the Shenzhou XII mission will be the first group tasked with building the nation's first space station－Tiangong, or Heavenly Palace.

39. In the first stage of the station's construction, astronauts on the Shenzhou XII and XIII missions and two cargo ships will be launched within a few months to prepare the module for docking with other parts of the station.

40. On October 15, 2003, China carried out its first manned space mission, sending Yang Liwei, a former fighter jet pilot, on a 21-hour journey around Earth in the Shenzhou V spacecraft.

41. After the core module is launched, astronauts on the Shenzhou XII and XIII missions and two cargo ships will be launched within a few months to prepare the module for docking with other parts of the station.

42. After this launch, astronauts in the Shenzhou XII and XIII missions and two cargo ships will be lofted within several months to prepare the module for its future docking with other parts of the station.

43. Space station module to be lofted in first half of 2021 "Zhang Xiaoguang, an astronaut on the 2013 flight of Shenzhou X, unveiled the mission's reentry capsule at a ceremony in Shaoshan, Hunan province, at which the capsule was given to the provincial government for permanent exhibition at the Mao Zedong Memorial Museum.

44. After the core module enters into orbit, China will launch the cargo spacecraft Tianzhou 2 and manned spacecraft Shenzhou XII.

45. The astronaut crew will remain in space for several months before the launch of Tianzhou 3 and Shenzhou XIII, which will send the second crew into space, Zhou added.

46. Zhou said exhibition of the Shenzhou X reentry capsule is meant to honor the forerunners of China's aerospace industry and integrate the spirit of space exploration into the revolutionary culture of Chairman Mao's hometown.

47. Zhou, also an academician of the Chinese Academy of Engineering, said he was very happy to see the Shenzhou X reentry capsule displayed at the museum.

48. Since Shuguang I, China has advanced greatly in space exploration, as shown in the Shenzhou series of successful crewed spaceflight missions, the Chang'e lunar exploration program and Tianwen 1, an interplanetary mission to Mars.

49. Shenzhou X was launched on June 11, 2013, carrying three astronauts, Nie Haisheng, Zhang Xiaoguang and Wang Yaping.

50. "Displaying the reentry capsule of Shenzhou X demonstrates that it was the growing, comprehensive power of our nation that created the Chinese space industry of today.

51. In fact, every successful space mission is a tribute to Chairman Mao and the old revolutionaries,"" he said."

52. China will launch Long March 5B carrier rocket into space in 2019, and after that, the rocket will help carry the core module of China's space station into space. Shenzhou XII and Shenzhou XIII spacecrafts will be launched soon after that.

53. But the crew of Shenzhou XII will consist of astronauts from the first and second batches, Yang said.

54. The three astronauts, whose sexes were not specified, will board the spacecraft Shenzhou IX to rendezvous and dock with Tiangong-1, an orbiting space lab, the spokesman said.

55. The mission will differ chiefly in one way from the automatic dockings that occurred between the unmanned spacecraft Shenzhou XIII and Tiangong-1 in November.

56. Made-in-Chengdu to help Shenzhou spacecraft return "The Shenzhou VIII spacecraft was launched under the spotlight at 5:58 am, Nov 1.

57. The controller, made by AVIC Chengdu CAIC Electronics Co Ltd, has been applied in Chinese spacecrafts from Shenzhou I through to Shenzhou XIII.

58. In addition, the company supplies two models of the pressure annunciator for Shenzhou VIII.

59. Shenzhou XIII crew readies for Lunar New Year in space Shenzhou XIII mission crew will spend the upcoming Spring Festival, or Chinese Lunar New Year in outer space, the first Chinese crew to spend the traditional holiday in space.

60. With the help of the Shenzhou spacecraft series, the Tiangong 2 space laboratory, and the Shijian 10 satellite, China has achieved mammalian embryonic development in space and in-orbit verification of the world's first space cold atom clock, expanded the understanding of the mechanisms behind particle segregation in microgravity, pulverized coal combustion, and material preparation, and achieved research findings in space science of international standing.

61. The Tianzhou 2 and Tianzhou 3 cargo spacecraft and the Shenzhou XII and Shenzhou XIII manned spacecraft, together with the Tianhe core module to which they have docked, form an assembly in steady operation.

62. Launch the Wentian and Mengtian experimental modules, the Xuntian space telescope, the Shenzhou manned spacecraft, and the Tianzhou cargo spacecraft;

63. TT&C missions of the Shenzhou and Tianzhou spacecraft series, Tianhe core module, Chang'e lunar probe series, and Tianwen-1 Mars probe have been completed successfully. TT&C station networks for commercial satellites are growing quickly.

64. The current six-month Shenzhou XIII mission by three taikonauts aboard the Tianhe core module is China's longest in manned space program.

65. Visit by mainland Olympic medalists boosts Macao's sports development "BEIJING - Shenzhou XIII crew members became the first Chinese to spend the nation's most important festival -- the Chinese Lunar New Year -- in outer space.

66. On Oct 16, 2021, the Shenzhou-13 mission sent the three taikonauts into the space station, for a six-month stay -- the longest-ever duration in the country's manned space program.

Astronauts

67. During their stay in Tiangong, the astronauts carried out two extravehicular activities, or spacewalks, using a large robotic arm and other equipment to install and adjust devices outside the station.

68. On Saturday three Chinese astronauts entered China's space station core module Tianhe, hours after the Shenzhou XIII spaceship was successfully launched on Saturday.

69. The astronauts entered Tiangong later that day after the two spacecraft docked with each other, becoming the first inhabitants of the station.

70. The astronauts entered Tiangong later that day.

71. Shenzhou XIII is scheduled to fly to Tianhe in October, carrying three astronauts for a six-month mission inside the core module.

72. China's Tiangong space station, which will house three astronauts for six months, will be a cozy home.

73. The astronauts for the Shenzhou XII mission are participating in intensive training, it said.

74. The astronauts had been inside the Tiangong space station for 21 days as of Friday afternoon.

75. The astronauts' host craft, the Tianhe core module, was lifted by a Long March 5B heavy-lift rocket at the Wenchang Space Launch Center in Hainan province on April 29.

76. Upon its completion, Tiangong will be manned regularly by groups of three astronauts in periods lasting several months.

77. At the beginning of the experiment, the astronauts in the core module teleoperated the Tianzhou 2 cargo craft to leave the front docking port of the core module's node cabin and move to the planed parking point, with the coordination of ground control engineers.

78. The first astronauts inside Tiangong－Major General Nie Haisheng, Major General Liu Boming and Senior Colonel Tang Hongbo－finished their 92-day mission in mid-September.

79. In October, a second group of three astronauts is scheduled to be sent to Tianhe in the Shenzhou XIII crewed spacecraft.

80. Nie and his crew members were tasked with testing and verifying plans, technologies and equipment for crucial elements of the Tiangong station's construction and operation, such as astronauts' long-term mission arrangements, the life-support system, in-orbit resupply, extravehicular activity and spacecraft maintenance and repair.

81. Yang Hong, the core capsule's chief designer at the China Academy of Space Technology, said Tianhe allows for astronauts' long-term stay and their extravehicular activities, which will be needed for them to assemble the station, examine its external condition or repair broken parts.

82. Chinese astronauts to host video call with students "Chinese astronauts on board the Tiangong space station hold a real-time video dialogue with Hong Kong students on Sept 3, 2021.

83. Three Chinese astronauts on the Tiangong space station interact with college students in Beijing via a livestream on New Year's Day.

84. From hosting a children's art gallery in space to answering questions about manned spaceflight, the three astronauts onboard China's Tiangong space station celebrated the New Year by cultivating science and inspiration in the country's youth.

85. The core module Tianhe provides astronauts six zones respectively for work, sleep, sanitation, dining, healthcare, and exercise.

86. The astronauts' spacecraft will dock with Tiangong's core capsule, which is scheduled to be lifted by a Long March 5B heavy-lift rocket from the Wenchang Space Launch Center in Hainan province in the coming weeks.

87. Astronauts to host call from Tiangong "Senior Colonel Wang Yaping juggles three apples as she floats in the Tiangong space station on Nov 24.

88. China will soon conduct its Shenzhou XIII manned space flight, sending three astronauts to stay in the country's Tiangong space station for six months.

89. The next crewed spaceflight－Shenzhou XIII－is scheduled to be launched in October at the Jiuquan center, sending another three astronauts to the Tiangong station to work there for six months.

90. China launched the Tianzhou 3 cargo spacecraft on Monday to deliver more supplies to the Tiangong space station and ensure that three astronauts who are scheduled to spend a record-breaking six months on it can live and work comfortably.

91. China plans to launch the Shenzhou XIII manned spacecraft early on Saturday morning, carrying three astronauts who will stay inside the country's Tiangong space station for six months, one of the lead officials in the country's manned space program said.

92. China plans to launch the Shenzhou XIII manned spacecraft early on Saturday morning, carrying three astronauts who will stay inside the country's Tiangong space station for six months, one of the lead officials in the country's manned space program said.

93. To accommodate the needs of the astronauts who will stay longer, Tianzhou 3 has carried 40 percent more drinking water than Tianzhou 2.

94. With the construction of the Tiangong space station, Chinese astronauts will also be able to stay in orbit for a long time and give lectures from space.

95. The module is central to the Tiangong station's construction and operations, given that astronauts will live there and control the entire station from inside.

96. Two cargo spacecraft, Tianzhou 2 and Tianzhou 3, already joined the space station earlier this year where the three astronauts will spend the next six months.

Space industry

97. Hidden heroes work hard in country's space industry "A photo of Deng Qingming in his spacesuit.

98. To explore the vast cosmos, develop the space industry and build China into a space power is our eternal dream,"" stated President Xi Jinping.

99. As many Chinese people celebrated the nation's latest spacewalk, in which Wang Yaping became China's first female spacewalker on Sunday evening and early Monday morning, they were also thinking of the heroes behind the great achievements that China's space industry has made.

90. Since 2016, China's space industry has made rapid and innovative progress, manifested by a steady improvement in space infrastructure, the completion and operation of the BeiDou Navigation Satellite System, the completion of the high-resolution earth observation system, steady improvement of the service ability of satellite communications and broadcasting, the conclusion of the last step of the three-step lunar exploration program (""orbit, land, and return""), the first stages in building the space station, and a smooth interplanetary voyage and landing beyond the earth-moon system by Tianwen 1, followed by the exploration of Mars.

91. Besides the astronaut videos, the Chinese space industry has provided more related materials to the public.

92. In May, after the nation's first interplanetary mission, Tianwen 1, successfully touched down on Mars, Xi sent a congratulatory letter calling it a new milestone in China's space industry.

93. The space industry helps to improve people's lives.

94. China's space industry is subject to and serves the overall national strategy.

95. China adopts a holistic approach in building its space industry.

96. China has formulated guidelines on commercializing its space industry.

97. China's space industry made several remarkable accomplishments last year.

98. While hailing the remarkable accomplishments of China's space industry, readers show their respect and gratitude toward those working hard to make this happen, as well as their best wishes for the three astronauts.

99. "Such tests are important in the space industry's effort to reduce the operational costs of spacecraft and help to realize an affordable, convenient method for people to make space trips,"" he said.

100. China's space industry serves its major strategic needs, and targets cutting-edge technology that leads the world.

1.The Chinese government has been proactive in developing the space industry, through policy measures and well-thought-out plans for space activities.

2. The space industry will continue to improve its integrated and open industrial system comprising system integrators, specialized contractors, market suppliers, and public service providers, and covering all links from research to production.

3. The space industry will contribute more to China's growth as a whole, to global consensus and common effort with regard to outer space exploration and utilization, and to human progress.

4. In the next five years, China's space industry will seize the opportunities presented by the expanding digital industry and the digital transformation of traditional industries, to promote the application and transfer of space technology.
