# Supplementary material for: Conceptual metaphors and image construction of China in the space probe reports of China Daily: a social cognitive approach
Source: Front Psychol. 2023 Jun 8;14:1202988. doi: 10.3389/fpsyg.2023.1202988 (PMC10286809; doi:10.3389/fpsyg.2023.1202988)
Supplement: Supplementary file 1 [file Data_Sheet_1.zip › supplementary material/tables and figures.docx]

Supplementary Material

# Supplementary Data

**Table 1 The main conceptual domains and the number of the metaphors.**

| Domains | Sub-domains(5135) | Words | Frequency(%) |
| --- | --- | --- | --- |
| Traditional culture | Traditional culture(1638) | Chang’e(1323); Yutu/Jade Rabbit(129); Shenzhou(69); Tiangong(58); Tianwen(41); Queqiao(18) | 31.90 |
| Strivers | Mission（1228） | mission(712); exploration(454); attempt(21); prototype(21)；endeavor(20) | 23.91 |
|  | Goal（103） | start(46); goal(42); dream(14); wake-up call(1) | 2.00 |
|  | Spirit（15） | spirit(15) | 0.29 |
| Significance | Achievements  （624） | success/successful/successfully(276); achieve/achievement(85); historic(82) ；breakthrough/breakthroughs(39);complete(35); strategic(30); feat(21)； contribute(20)；accomplish/accomplishment(17)； fulfill(15)；highlight(4) | 12.15 |
|  | Power（181） | lift(108); power(72)；emphatic(1) | 3.52 |
|  | Action（5） | leap(5) | 0.10 |
|  | Fruit（3） | fruit(3) | 0.06 |
| Time | Time period（301） | develop(295); upcoming(6) | 5.86 |
|  | Point in time（32） | era(17); moment(14); timeline(1) | 0.62 |
| Journey | Trip（656） | Long March(427); step(76); phase(71); stage(53); course(15); on its way/pave the way(14) | 12.78 |
|  | Landmark(45) | milestone(24); landmark(19); roadmap(2) | 0.88 |
| Difficulty | Difficulty (121) | challenge/challenging(66); complicated(41) ; overcame(14) | 2.36 |
| Family | Family(69) | home(39); backup(20); safely(8); homecoming(2) | 1.34 |
| Leadership | Leadership (62) | heads(53); lead(9) | 1.21 |
| Share | Share(32) | share(29); willingness(3) | 0.62 |
| Chapter | Chapter(16) | chapter(16) | 0.31 |
| Music | Music(4) | trilogy(2); rehearsal(2) | 0.08 |

**Table 2 The main conceptual domains and the number of the metaphors.**

| Domains | Sub-domains(835) | Words | Frequency(%) |
| --- | --- | --- | --- |
| Traditional culture | Traditional culture(520) | Shenzhou(295)； Tiangong(108); Tianhe(52); Tianzhou(48)； Tianwen(7); Wentian(3)；Mengtian(3); Chang’e(3); culture(1) | 62.28 |
| Strivers | Mission(153) | mission(144); exploration(9) | 18.32 |
|  | Goal(2) | spirit(1); proactive(1) | 0.24 |
|  | Spirit(1) | dream(1) | 0.12 |
| Significance | Meaning(57) | success/successful/successfully(22); complete(21); remarkable(2); historic(2); improve/improvement(4); revolutionary(1)；strategic(1); advanced(1)； promote(2)； cutting-edge(1) | 6.83 |
|  | Achievement(26) | accomplish/accomplishment(17)；achieve/achievement(5);feat(1)； contribute(2)； tribute(1); | 3.11 |
|  | Action(3) | stride(2); seize(1) | 0.36 |
| Journey | Trip(53) | Long March(27); journey(13); travel(4); trip(4); voyage(1); level(1)； foundation(2); closed the gap(1) | 6.31 |
|  | Occupants（2） | occupants(2) | 0.24 |
|  | Landmark(1) | milestone(1) | 0.12 |
| Family | Famliy(10) | home(5); backup(5) | 1.20 |
| Leadership | Lead(3) | leading(1); guidelines(1); forerunners(1) | 0.36 |
|  | Hero（3） | hero(3) | 0.36 |
| Time | Point in time（1） | age(1) | 0.12 |

# Supplementary Figures and Tables

## Supplementary Figures

**
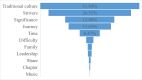
**

**Figure 1.** **The macro-level sematic domains of conceptual metaphors**

**
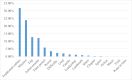
**

**Figure 2** **The micro-level sematic domains of conceptual metaphors**

**
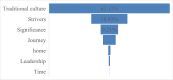
**

**Figure 3**  **The macro-level sematic domains of conceptual metaphors**

**
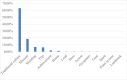
**

**Figure 4**  **The micro-level sematic domains of conceptual metaphors**
